# Supplementary material for: The interactome of intact mitochondria by cross-linking mass spectrometry provides evidence for coexisting respiratory supercomplexes
Source: Mol Cell Proteomics. 2017 Dec 8;17(2):216–32. doi: 10.1074/mcp.RA117.000470 (PMC5795388; doi:10.1074/mcp.RA117.000470)
Supplement: Supplemental Data [file supp_RA117.000470_133922_0_supp_23978_fzffwf.zip › spectra_annotation/mito_DR_spectra_annotation/30-1-2-1-1-1.pdf]

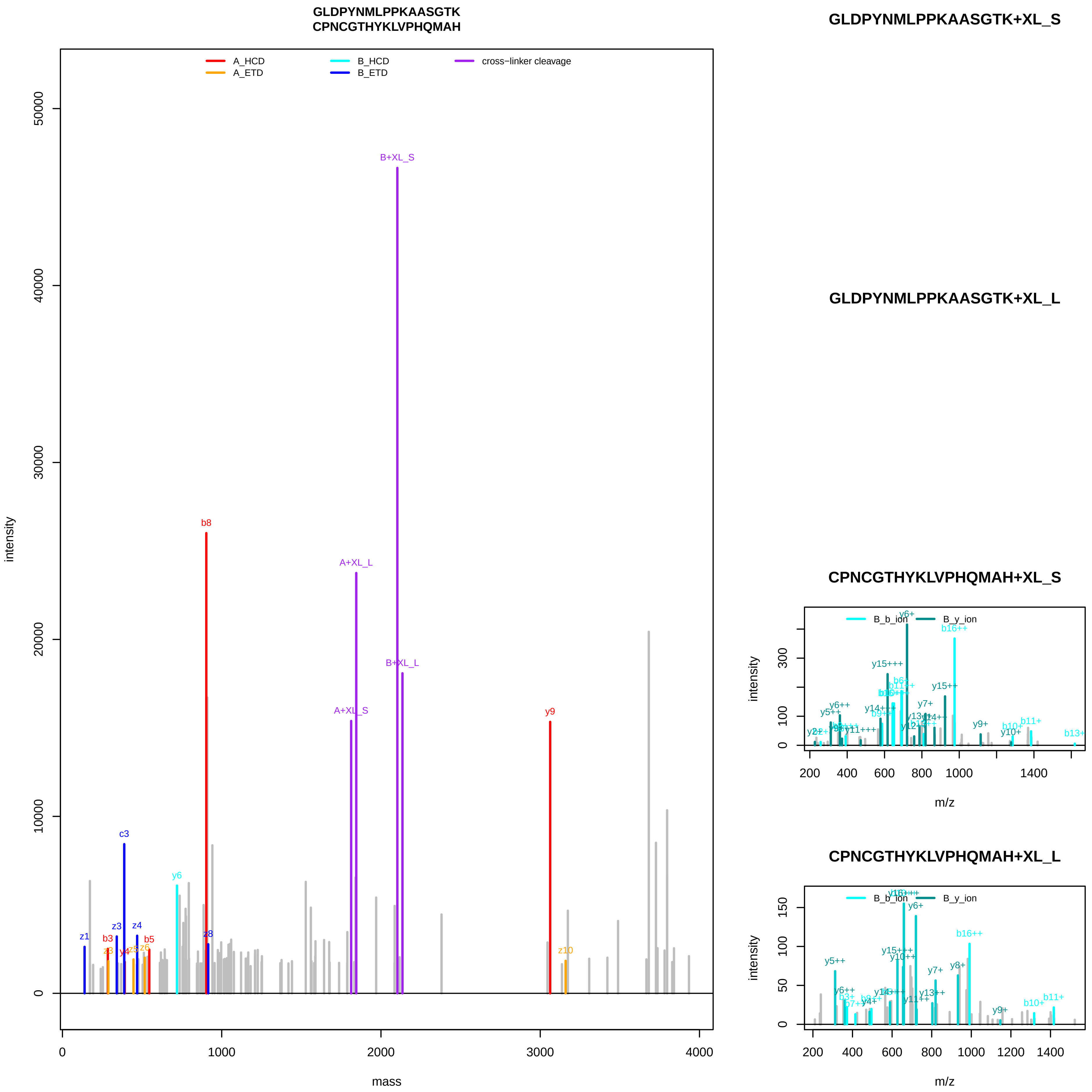
GLDPYNMLPPKAASGTK
CPNCGTHYKLVPHQMAH
A_HCD
A_ETD
B_HCD
B_ETD
cross−linker cleavage
B+XL_S
A+XL_L
B+XL_L
A+XL_S
b8
y9
c3
y6
z1
b3
z3
z4
b5
z8
z10
intensity
mass
0
10000
20000
30000
40000
50000
1000
2000
3000
4000
GLDPYNMLPPKAASGTK+XL_S
GLDPYNMLPPKAASGTK+XL_L
CPNCGTHYKLVPHQMAH+XL_S
B_b_ion
B_y_ion
y6+
b16++
y15+++
y15++
y7+
y9+
y10+
b10+
b11+
b13+
y5++
y6++
intensity
m/z
200
400
600
800
1000
1400
100
300
CPNCGTHYKLVPHQMAH+XL_L
B_b_ion
B_y_ion
y6+
b16++
y15+++
y10++
y7+
y8+
y13++
y11++
y9+
b10+
b11+
y5++
y6++
intensity
m/z
50
100
150
1200
